# Identification and comparison of *Chlamydia psittaci* , *Legionella* and *Mycoplasma pneumonia* infection

**DOI:** 10.1111/crj.13603

**Published:** 2023-03-17

**Authors:** Ning Zhu, Daibing Zhou, Ruyu Yuan, Yiminniyaze Ruzetuoheti, Jing Li, Xiujuan Zhang, Shengqing Li

**Affiliations:** ^1^ Department of Respiratory and Critical Care Medicine, Huashan Hospital Fudan University Shanghai China

**Keywords:** *Chlamydia psittaci*, community‐acquired pneumonia (CAP), *Legionella*, metagenomic next‐generation sequencing (mNGS), *Mycoplasma*, treatment

## Abstract

**Introduction:**

Conventional etiological detection and pathogenic antibody methods make it challenging to identify the atypical pathogens among the community‐acquired pneumonia (CAP). Metagenomic next‐generation sequencing (mNGS) could rapidly detect all potentially infectious diseases and identifies novel or potential pathogens.

**Methods:**

Eighteen patients diagnosed with atypical CAP were enrolled in this retrospective study, including nine *Chlamydia psittaci pneumonia* (*C. p*), four *Legionella pneumonia* (*L. p*) and five *Mycoplasma pneumonia* (*M. p*). We simultaneously tested bronchoalveolar lavage fluid (BALF) samples for conventional microbiological methods and mNGS, and blood specimens were analysed. We also collected and compared baseline and clinical characteristics and treatment responses.

**Results:**

Patients with *C. p* and *L. p* had similar symptoms, including fever, cough, headache, dyspnoea, asthenia, shivering and headache, compared with *M. p*, whose symptoms were slight. *C. p* and *L. p* usually showed multiple lobar distributions with pleural effusion. Serologic testing indicated that *L. p* had higher levels of white blood cells (WBCs), neutrophils, C‐reactive protein (CRP), procalcitonin (PCT), alanine aminotransferase (ALT), lactate dehydrogenase (LDH) and creatinine compared with *M. p* and *L. p* (*p* < 0.05). However, patients with *C. p* had lower levels of albumin (*p* < 0.05), and *M. p* had a minimum risk of cardiac volume loads (*p* < 0.05). CD4/CD8 ratio, lymphocytes, aspartate aminotransferase (AST), creatine kinase (CK), cell counting of BALF and coagulation had no difference (*p* < 0.05). Pathogenic IgM assay showed that 4/5 cases were positive for *M. p* and no positive detection for *C. p* and *L. p* infection. We timely adjusted the antibiotics according to the final mNGS results. Eventually, 16/18 patients recovered fully. Conditions of *L. p* patients were worse than those of *C. p* patients, and those of *M. p* patients were the least.

**Conclusion:**

Early application of mNGS detection increased the atypical pathogenic identification, improved the prognosis and made up for the deficiency of conventional detection methods.

## Graphical abstract

In this retrospective study, all the atypical pathogens including *Chlamydia psittaci* , *Legionella* and *Mycoplasma* were identified by mNGS. A comparison of clinical characteristics of nine *Chlamydia psittaci pneumonia* (*C. p*), four *Legionella pneumonia* (*L. p*) and five *Mycoplasma pneumonia* (*M. p*) were firstly accessed.

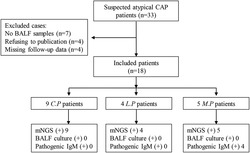

## 1 INTRODUCTION

Community‐acquired pneumonia (CAP) is a common infectious cause of morbidity and mortality worldwide. It is a heterogeneous disease with clinical manifestations, illness severity and diverse pathogens.^1^ *Mycoplasma pneumoniae* (*M. p*), *Chlamydiae psittaci* pneumoniae (*C. p*) and *Legionella* spp *pneumoniae (L. p)* are common causes in atypical pneumonia among immune‐competent hosts.^2^ Patients with *M. p* infection usually occur in school‐aged children and young adults. It occurs primarily in crowded settings where people interact for a long time.^3^ As with *M. p*, *C. p* also occurs in a closed area because of prolonged interaction, like school and nursing homes. Patients with *L. p* usually have been infected through exposure to humid‐contaminated settings, especially for immunosuppressed, immunocompromised and older adults, as well as those with a history of smoking.^4^, ^5^ The common symptoms caused by *M. p*, *C. p* and *L. p* are nonspecific from the mild to the severe, including fever, chills, sneezes, cough, phlegm, shortness of breath and tiredness, with some extrapulmonary manifestations.^3^ Although radiological imaging plays an essential role in diagnosing atypical pneumonia, the variable manifestations of *C. p*, *M. p* and *L. p*, including perihilar ground‐glass opacities, unilateral or multilobar consolidation, are widely variable and atypical. The ipsilateral presence of pleural effusion with multilobar consolidation often occurs in *C. p* and *L. p*, and lymphadenopathy is uncommon.^2^, ^3^, ^6^

The preferred diagnostic tests for *C. p*, *M. p* and *L. p* are the culture of low respiratory samples and serological tests.^1^ For example, the serological evidence of *C. p* was as follows (at least one item): at least four‐fold higher than the upper limit of normal in duplicate serum samples; the titre of IgM antibody is 1:16 by micro‐immunofluorescence (MIF) assay.^7^ As for *M. p*, recommended identifications of *L. p* were the isolation of *Legionella* by culture and *Legionella* urinary antigen. A systematic review showed that the sensitivity and specificity of *Legionella* urinary antigens were 0.74 and 0.991, respectively.^8^ Regarding the laboratory tests of *M. p*, the culture of respiratory, serology and nucleic acid amplification methods were available.^9^ However, identifying atypical bacteria like *C. p*, *M. p* and *L. p* infection by conventional culture could be time‐consuming and difficult. In addition, we usually require serological antibodies to collect acute and convalescent paired serum samples. Multiple testing of CAP patients is applied to reduce the diagnostic deficit and under‐ascertainment.

In recent years, metagenomic next‐generation sequencing (mNGS) has been applied in all potentially infectious diseases and identifies novel or potential pathogens, regardless of microorganism species.^10^ mNGS allows thousands to billions of DNA fragments to be independently sequenced simultaneously. Compared with conventional tests, mNGS has high‐throughput capacity and short test time characteristics. mNGS can detect particular microorganisms such as mycobacteria and fungi, which need more time, up to weeks, for insidious pathogens. Sequencing depths usually coincided with more microbial reads.^11^ Therefore, mNGS can help identify etiologic pathogens and provide a prediction of drug resistance.^12^

In this study, we performed a retrospective analysis of 18 cases of CAP diagnosed by mNGS, including nine *C. p*, five *M. p* and four *L. p*. We analysed their epidemiology, clinical manifestations, radiology and serological results and mNGS results of bronchoalveolar lavage fluid (BALF) samples as well as the outcome.

## 2 METHODS

### 2.1 Study design and participants

**FIGURE 1:**
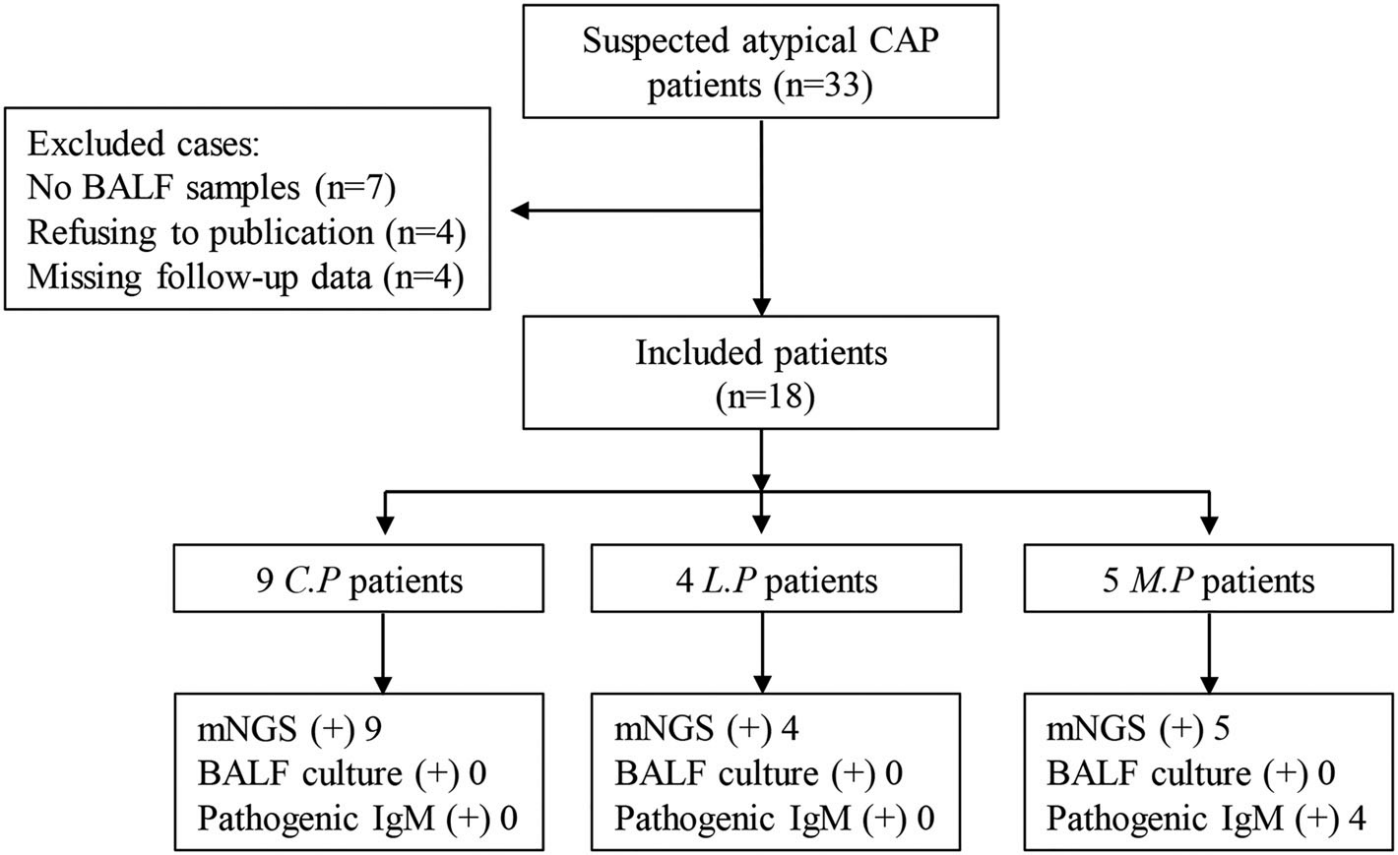
Flow chart of the study. CAP, community‐acquired pneumonia; BALF, bronchoalveolar lavage fluid; *C.P*, *Chlamydia psittaci* pneumonia; *L.P*, *Legionella pneumonia*; *M.P*, *Mycoplasma pneumonia*; mNGS, metagenomic next‐generation sequencing.

A total of 33 patients with suspected atypical CAP were initially enrolled from November 2018 to December 2021 at Huashan Hospital, Fudan University (Figure 1). Fifteen cases were excluded for no BALF samples (seven), refused to publication (four) and missing follow‐up data (four). Finally, 18 CAP patients with atypical pathogens were included if they met the following criteria: (1) age ≥ 18 years, (2) diagnosed with atypical CAP according to the guidelines,^1^, ^13^ (3) positive result of an atypical pathogen from BALF sample or serum pathogenic IgM antibody and (4) chest CT showing reduced or disappeared changes within 2 weeks after effective treatment. Exclusion criteria were as follows: (1) no BALF samples for mNGS and (2) BALF samples collected more than 72 h from admission to the hospital. This study was approved by the Ethics Committee of Huashan Hospital, Fudan University (NO.KY2016‐396). All patients gave written informed consent.

### 2.2 Clinical data collection and treatment

All the epidemiology, clinical manifestations, mNGS results, laboratory, outcome data and CURB‐65 scores were retrieved from the medical records. We finished the follow‐up on 30 January 2022. Patients had undergone empirical antibiotic treatment according to the guidelines.^1^, ^13^ Enrolled cases underwent bronchoscopes after adequate preoperative assessments. BALF samples were collected for mNGS analysis and conventional tests and obtained for airway cell enumeration. Antibiotic strategies were adjusted based on the conventional tests or mNGS results combined with the inflammatory biomarkers and radiology.

### 2.3 mNGS and data analysis

An experienced technician performed DNA extraction, library construction and sequencing. All specimens were promptly stored in sterile pipes. Briefly, BALF samples were centrifuged, homogenized and subjected to DNA extraction using TIANamp Micro DNA Kit (DP316, TIANGEN Biotech). DNA libraries were constructed by DNA fragmentation, end‐repair, adapter ligation and PCR amplification and subjected to quality control analysis using Agilent 2100 bioanalyzer. Quality‐verified libraries were sequenced by the BGISEQ‐50 platforms (BGI Genomics, Shenzhen). After filtering low‐quality reads, human host sequences and low‐complexity reads, taxonomy assignment was conducted by sequence alignment using Burrows–Wheeler alignment to reference databases.^14^ Negative control was included in each run, and internal control was added to every sample. Each final report of detected pathogens was reviewed and proofread by another technician.

### 2.4 Statistical analysis

All analyses were performed with SPSS Statistics 21 (SPSS, Chicago, IL, USA). Continuous variables were described as median with interquartile range and categorical variables as frequency. A nonparametric Wilcoxon rank‐sum test was applied to compare the difference between groups. A *p*‐value of <0.05 was considered significant.

## 3 RESULTS

### 3.1 Patients and clinical characteristics

Demographic characteristics of 18 cases are provided in Table 1. Of the 18 cases, 13 were males and 5 were females. There was no difference in BMI index and age among *C. p*, *L. p* and *M. p* (*p* > 0.05) (Table 1). 6/9 of *C. p* had a history of cigarette exposure (more than 5 years), 3/4 in *L. p* and 1/5 in *M. p*. Nine patients had a history of COPD (six in *C. p*, three in *L. p* and 0 in *M. p*). Of four *L. p* cases, one patient had a positive result of T‐SPOT.TB assay. 18/18 cases had no history of HBV infection. As seen in Table 2, 5/9 patients with *C. p* (P1 to P9) had exposure to birds, bird droppings or live poultry market. Only one *L. p* patient who worked for long‐distance transport used to live in small hotels.

**TABLE 1:** Baseline characteristics of patients on admission.

Characteristics	*C. p* (*n* = 9)	*L. p* (*n* = 4)	*M. p* (*n* = 5)	*χ* ^2^	*P*‐value*
Demographics					
Female/Male	6/3	4/0	3/2		
Age (years)	73 (40–80)	52.5 (33–63)	42 (14–81)	4.019	0.134
BMI	20.28 (15.94–27.55)	27.43 (23.67–29.38)	23.23 (15.47–26.4)	5.072	0.079
History					
Cigarette (≥5 years)	6	3	1		
COPD	6	3	0		
HBsAg (−)	9	4	5		
T‐SPOT.TB (−)	9	3	5		
Clinical manifestations					
Fever ≥ 39°C	8	4	1		
Cough	9	4	5		
Phlegm	9	0	1		
Dyspnoea	7	4	2		
Asthenia	7	3	5		
Shivering	8	4	1		
Headache	6	4	1		
Diarrhoea	2	3	0		
Abdominal distension or pain	2	3	0		
CURB‐65 scores	1.0 (1.0–5.0)	2.5 (2.0–4.0)	0.0 (0.0–2.0)	4.938	0.085
Chest image					
Unilateral patchy diffusions	3	0	3		
Multiple lobar distributions	6	4	2		
Pleural effusion	5	4	0		
Blood testing (normal range)					
CD4/CD8 ratio (0.9–3.6)	2.34 (1.59–6)	1.87 (1.2–2.78)	1.8 (1.24–2.36)	4.339	0.114
WBC (3.5–9.5 × 10^9/L)	8.5 (3.72–15.52)	11.1 (9.11–12.39)	5.98 (3.89–6.88)	6.459	**0.040**
Neutrophils (1.8–6.3 × 10^9/L)	7.78 (3.52–12.27)	9.68 (7.23–10.53)	4.74 (1.91–6.23)	6.340	**0.042**
Lymphocytes (1.1–3.2 × 10^9/L)	0.51 (0.12–1.76)	0.93 (0.69–1.49)	1.19 (0.70–1.64)	5.604	0.061
CRP (0–5 mg/L)	188 (116.3–284.53)	224.4 (211–400.19)	27.5 (1.34–308.5)	6.938	**0.031**
PCT (0–0.05 ng/mL)	1.18 (0.16–3.1)	2.49 (0.68–6.29)	0.48 (0.03–0.52)	6.474	**0.039**
ALT (19–50 U/L)	84 (28–124)	116.5 (104–145)	52 (15–103)	7.366	**0.025**
AST (15–40 U/L)	97 (46–264)	210 (40–346)	32 (19–153)	4.316	0.116
Albumin (40–55 g/L)	30 (25–34)	36.5 (27–43)	36 (29–45)	6.153	**0.046**
LDH (120–250 U/L)	413 (274–695)	571.5 (469–1897)	43 (34–664)	6.474	**0.039**
CK (50–310 U/L)	216 (22–1092)	647 (142–3354)	43 (34–664)	2.363	0.307
Pro‐BNP (0–100 pg/mL)	664.6 (195.5–4669)	190.5 (123.3–703.2)	224.3 (87.3–332.5)	7.989	**0.018**
Creatinine (57–110 μmol/L)	66 (46–85)	94 (82–137)	65 (51–93)	6.784	**0.034**
D‐dimer (0–0.5 mg/L)	1.68 (1.27–3.88)	3.55 (1.07–5.45)	1.41 (1.07–12.14)	0.514	0.773
APTT (20.3–32.3 s)	32.5 (25–52.7)	34.9 (28.2–36.5)	28.5 (21.1–42.2)	1.335	0.513
PT (10.7–13 s)	13.5 (12.5–14.2)	13.7 (13.4–14.7)	12.7 (10.6–15.4)	3.009	0.222
BALF testing					
Neutrophils (%)	72 (38–93)	62 (20–76)	71 (16–85)	1.400	0.497
Lymphocytes (%)	20 (2–62)	36 (23–76)	29 (15–84)	2.083	0.353

**TABLE 2:** Clinical information and BALF results of conventional culture and mNGS.

No.	Gender/age	History of contact	CURB‐65 scores	Pathogenic IgM	Conventional culture (BALF)	mNGS results	
						Genus (reads)	Species (reads)
P1	M/67	Birds	5	(−)	(−)	*Chlamydia* (54)	*Chlamydia psittaci* (38)	
P2	M/57	Bird droppings	1	(−)	(−)	*Chlamydia* (3961)	*Chlamydia psittaci* (2891)
P3	M/80	No	4	(−)	(−)	*Chlamydia* (3920)	*Chlamydia psittaci* (2298)
P4	F/53	Live poultry market	1	(−)	(−)	*Chlamydia* (867)	*Chlamydia psittaci* (538)
P5	F/40	No	1	(−)	(−)	*Chlamydia* (6)	*Chlamydia psittaci* (6)
P6	M/73	No	3	(−)	*Aspergillus*	*Chlamydia* (2151)	*Chlamydia psittaci* (1402)
P7	F/75	Live poultry market	1	(−)	(−)	*Chlamydia* (1)	*Chlamydia psittaci* (1)
P8	M/75	No	1	(−)	(−)	*Chlamydia* (9)	*Chlamydia psittaci* (5)
P9	M/80	Bird droppings	3	(−)	*Candida albicans*	*Chlamydia* (176)	*Chlamydia psittaci* (114)
P10	M/33	hotel	4	(−)	(−)	*Legionella* (2)	*Legionella pneumophila* (2)
P11	M/63	No	3	(−)	(−)	*Legionella* (2246)	*Legionella pneumophila* (1,917)
P12	M/61	No	2	(−)	*Candida tropicalis*	*Legionella* (117)	*Legionella pneumophila* (110)
P13	M/44	No	2	(−)	(−)	*Legionella* (59)	*Legionella pneumophila* (55)
P14	M/14	No	0	*Mycoplasma* (+weak)	*Candida albicans*	*Mycoplasma* (8746)	*Mycoplasma poneumoniae* (8704)
P15	M/19	No	0	*Mycoplasma* (+)	(−)	*Mycoplasma* (26)	*Mycoplasma poneumoniae* (25)
P16	M/42	No	0	*Mycoplasma* (+)	(−)	*Mycoplasma* (50)	*Mycoplasma poneumoniae* (50)
P17	F/48	No	2	(−)	(−)	*Mycoplasma* (6)	*Mycoplasma poneumoniae* (6)
P18	F/81	No	2	*Mycoplasma* (+)	(−)	*Mycoplasma* (5)	*Mycoplasma poneumoniae* (5)

Clinical manifestations are shown in Table 1. On admission, more than half of *C. p* patients had a fever over 39°C (8/9, 88.89%), cough (9/9, 100%), phlegm (9/9, 100%), dyspnoea (7/9, 77.78%), asthenia (7/9, 77.78%), shivering (8/9, 88.89%) and headache (6/9, 66.67%), and only 2/9 patients had diarrhoea and abdominal distension or pain. The median of CURB‐65 scores was 1.0 for *C. p* (range 1–5). As with *C. p* patients, the complaints of *L. p* cases presented with intra‐ and extra‐pulmonary fever (≧39°C) (4/4, 100%), cough (4/4, 100%), dyspnoea (4/4, 100%), asthenia (3/4, 75%), shivering (4/4, 100%), headache (4/4, 100%), diarrhoea (3/4, 75%) and abdominal distension or pain (3/4, 75%), and phlegm was uncommon. The median of CURB‐65 scores was 2.5 (range 2–4). Unlike *C. p* and *L. p* patients, these symptoms were typically mild for *M. p*; they usually exhibited cough (5/5, 100%) and asthenia (5/5, 100%). 1/5 patients had a fever, phlegm and shivering, and 2/5 exhibited dyspnoea. We did not observe gastrointestinal symptoms like diarrhoea, abdominal distension or pain. Overall, the symptoms of *M. p* patients were mild, and CURB‐65 scores were 0 (range 0–2).

### 3.2 Radiological images and laboratory tests

On admission, among nine *C. p* patients, chest CT imaging at the early stage of infection revealed consolidation with high density (3/9 in one lobe, 6/9 in multiple lobar) and mild to moderate pleural effusion in six patients. We observed that all the *L. p* patients had radiological imaging of multiple lobar distributions with pleural effusion (4/4). Patients with *M. p* performed unilateral patchy diffusions (3/5) and multiple lobar distributions (2/5), but pleural effusion was uncommon.

Laboratory results are detailed in Table 1. Serum and BALF samples were obtained for the first 24 h on admission. Of the 18 patients, the radio of CD4/CD8 indicated the normal range among three microorganism infections (*p* > 0.05), and it reflected almost identical non‐immunosuppressive states. Patients with *L. p* exhibited elevated white blood cell (WBC) and neutrophils level (median 11.1 × 10^9/L and 9.68 × 10^9/L, respectively) compared with *C. p* (median 8.5 × 10^9/L for WBC and 7.78 × 10^9/L for neutrophils, respectively) and *M. p* (median 5.98 × 10^9/L for WBC level and 4.72 × 10^9/L for neutrophils, respectively) (*p* < 0.05). *M. p* showed a relatively low level of WBC and neutrophils within the normal range. Interestingly, 13/18 patients (nine cases of *C. p* and four *L. p*) exhibited decreased lymphocytes (median value 0.51 × 10^9/L and 0.93 × 10^9/L, respectively), both below the standard lower limit. Cases of *M. p* patients had no decreased level of lymphocytes (median value 1.19 × 10^9/L). However, levels of neutrophils in the *L. p* group did not increase statistically in the BALF testing (*p* = 0.497); the same results applied to lymphocytes (*p* = 0.353). All patients exhibited elevated C‐reactive protein (CRP) and procalcitonin (PCT) levels; *L. p* cases were the highest, *C. p* the second and *M. p* the third (*p* < 0.05). Eighteen cases of patients had a different level of liver function abnormality with alanine aminotransferase (ALT) above the normal range, but the aspartate aminotransferase (AST) in the normal range (Table 1). As compared with the group of *M. p*, ALT levels were found to be elevated by two‐fold in the *L. p* group (median value 116.5 U/L) and 1.5‐fold in the *C. p* group (*p* = 0.025). Levels of albumin decreased to near 10 g/L in the *C. p* group. In contrast, it reduced near 4 g/L below the lower limit in the *L. p* and *M. p* group (*p* = 0.046). Most patients also had abnormal myocardial zymograms, presenting elevated lactate dehydrogenase (LDH) in the *L. p* and *C. p* groups, compared with the *M. p* group (*p* = 0.039). Creatine kinase (CK) levels had no apparent significance in these three groups (*p* = 0.307). However, the serum CK levels increased twice higher above the upper limit. All patients had high N‐terminal‐pro hormone BNP (pro‐BNP) expression, representing abnormal cardiac volume load, especially in the *C. p* group (*p* = 0.018). Although the median creatinine values remained within the normal limits, the *L. p* group patients were inclined to increase the risk of renal damage. In addition, the data revealed that the serum level of D‐dimer, APTT and PT had no apparent changes in all groups (*p* > 0.05).

The final report of mNGS took 48–72 h from the receipt of the sample. Distribution and classification of mNGS results, conventional culture and IgM antibody for pathogens were presented in Table 2. MIF tests nine respiratory IgM antibodies of pathogens, including *Legionella pneumonia type 1*, *Mycoplasma pneumonia*, *rickettsia Q*, *Chlamydia pneumonia*, *adenovirus*, *respiratory syncytial virus*, *influenza A/B virus* and *parainfluenza virus types‐1/‐2/‐3*. Of five patients in *M. p*, four cases showed positive staining of IgM antibody for *Mycoplasma*. No positive antibody cases were observed in *Chlamydia* or *Legionella pneumonia type 1*, both in the *C. p* and *L. p* groups.

All patients underwent fibre bronchoscopy within 24 h of admission. Samples based on BALF were tested for mNGS and routine microbiology. The mNGS test revealed positive results, including nine cases of *C. psittaci*, four *Legionella* and five *Mycoplasma*. However, routine microbiology testing had no positive results for *C. psittaci*, *Legionella* and *Mycoplasma* (Table 2).

### 3.3 Treatment and outcomes

All patients received antibiotics treatments, including cephalosporin or penicillin combined with quinolones. If patients' condition deteriorated and severe respiratory failure developed, patients underwent mechanical ventilation. As shown in Table 3, of nine *C. p* patients, one underwent nasal catheter oxygen inhalation, six for high flow humidified oxygen therapy and two for invasive mechanical ventilation (IMV). 2/4 cases required high‐flow oxygen inhalation and 1/4 for IMV in the *L. p* group. All *M. p* patients only received nasal catheter oxygen inhalation. When *C. p*, *L. p* and *M. p* infections were established, the antibiotic therapy was readjusted to quinolones or minocycline for at least 2 weeks. Eventually, 16 patients experienced full recoveries. One *C. p* patient died from secondary multidrug‐resistant Klebsiella and mixed aspergillus infection, which caused a progressive decrease in septic shock and led to death during the hospitalization. In addition, another *C. p* patient died because of accidental injury within 30 days of discharge. No mortalities occurred before the end of the follow‐up.

**TABLE 3:** Treatment and outcomes.

	*C. p* (*n* = 9)	*L. p* (*n* = 4)	*M. p* (*n* = 5)
Oxygen therapy			
Nasal catheter	1	1	5
High flow	6	2	0
Invasive mechanical ventilation	2	1	0
In‐hospital mortality	1	0	0
30‐day mortality	1	0	0

## 4 DISCUSSION

Our retrospective study of 18 patients with atypical CAP shed light on the emerging application of mNGS in detecting atypical pathogens by comparing the pathogenic diagnosis of conventional testing, pathogenic IgM antibody and mNGS. We have established that mNGS could help us identify unique and rare pathogens within 48–72 h and adjust personalized antibiotic treatment quickly. Because of extensive empiric antibiotics, pathogenic culture time‐consuming and variations in patients' conditions, there were limitations of conventional pathogenic tests. In our study, the conventional test did not detect atypical pathogenic microorganisms like *C. p*, *L. p* and *M. p*. Although we observed four positive pathogenic IgM antibody cases for *M. p* by MIF assay, it could not provide enough of the load and subtype of pathogens.

In the present study, we observed that *C. psittaci* and *Legionella* infections could cause a more pronounced inflammatory response, including increased WBC, neutrophils, CRP and PCT, especially in patients with *L. p*, both compared with *Mycoplasma* infection. On admission, a *Legionella* infection (P10) case had a high fever of 39.6°C, shortness of breath and hypersomnia. He exhibited blood CRP levels of 220 mg/L and PCT of 6.25 mg/L, and CURB‐65 scores were five. He was undergone mechanical ventilation within 36 h. It also reported that a patient with *C. psittaci* infection who manifested with multiple organ dysfunction and a high level of PCT (6.51 ng/mL) underwent extracorporeal membrane oxygenation (ECMO).^15^ Because *C. psittaci* and *Legionella* are difficult to culture and highly infectious, experts advised a test of IgM antibody assay by MIF for *C. psittaci IgM* ≧ 1:16 and *Legionella IgM* ≧ 1:128 in acute and convalescent sera.^8^ A systemic review showed that *Legionella* urinary antibody assay had a sensitivity of 0.74 and specificity of 0.991.^16^ However, laboratory conditions and lower loads of bacteria prevented the technique from popularising. All existing factors led to a higher possibility of *C. psittaci* and *Legionella* infections developing sepsis and septic shock, especially accompanying high PCT levels.^17^ Pneumonia due to *Mycoplasma* infection mainly presented with mild upper respiratory symptoms and less severe pulmonary manifestations.^3^ In our study, 5/5 patients had cough without phlegm and asthenia, 2/5 had dyspnoea and 1/5 had a high fever and slight liver dysfunction, making it difficult to distinguish from other respiratory infections.

Radiologic chest images due to *Chlamydophyla*, *Legionella* and *Mycoplasma* infections seem to be a diagnostic challenge. Radiologic findings usually show *C. p* infection presented with a single lobe with lower lobe involvement.^3^ In addition, patients with *C. p* infection showed pulmonary consolidation, broncho‐vascular bundle thickening, ground‐glass opacities and small‐ to moderate‐sized homolateral pleural effusion by chest CT findings.^3^ Legionnaires' disease is similar to other typical and atypical pneumonia. More than one lobe consolidation in chest CT is a common imaging manifestation of *L. p*.^3^, ^8^ The early radiographs of 159 cases usually manifested as airspace consolidation of lower lobes, pleural effusion was rare, and no pneumothorax or cavitation.^18^ However, we should consider the CT images of *L. p* infection as a differential diagnosis of pulmonary nodules and pleural empyema in immunocompromised patients.^19^, ^20^ Patients with *L. p* admitted to the ICU presented with multiple lobes diffusion, compared with *C. p*.^21^ Imaging features of *M. p* often showed bronchopneumonia, extensive unilateral and bilateral lung consolidation, pleural effusion and atelectasis in paediatric patients.^22^, ^23^, ^24^ In our study. The median age of four cases with *M. p* was 42 years (range 14–81), and three cases were more than 18 years old. 3/5 patients showed unilateral diffusion, and 2/5 had slight to moderate pleural effusion, making it difficult to distinguish from other atypical bacteria.

The clinical features and radiographic images of CAP caused by atypical pathogens usually overlap with those of other ‘typical’ CAP, such that the guideline of CAP would not distinguish the difference between different atypical pathogens, resulting in potentially misleading and even missing the optimal timing of treatment. Further optimization of test conditions will be required to improve diagnostic values. The strategy of mNGS detection can effectively overcome the insufficiency. The sensitivity and specificity of mNGS were 50.7% and 85.7% among infectious diseases, respectively.^25^ In our previous study, we found that the application of mNGS could improve the pulmonary TB identifications with a sensitivity of 89.13% and specificity of 98.36%.^26^ A study showed that CAP patients' overall microbial detection rate was 90.3% for mNGS versus 39.5% for conventional tests.^27^ mNGS can detect microorganisms from samples like BLAF, cavity effusion, biopsy tissue, cerebrospinal fluid, urine, blood and sputum.^26^, ^28^, ^29^, ^30^ The application of mNGS for immunocompromised CAP patients could provide a wide range of potential pathogens and improve the prognosis.^30^ Haibing Liu and his colleagues found that several antibiotic resistance genes of bacteria by mNGS were consistent with drug sensitivity test.^12^ Nowadays, mNGS provide a new insight for a promising pathogenic identification for various infectious disease and is further likely to evaluate immunological competence to offending agents, virulence genes and more antimicrobial genes. Our study had certain limitations: (1) small cases were enrolled, (2) the restriction of *Legionella* urinary antigen test in the hospital and (3) the samples had inevitable heterogeneity.

In conclusion, although patients with *C. p* and *L. p* had similar symptoms and showed multiple lobar distributions with pleural effusion, *L. p* patients were likely to develop respiratory failure and organ function abnormalities like liver and kidney. Compared with *C. p* and *L. p*, symptoms and chest CT imaging were slight for *M. p* cases. Clinical heterogeneity and radiologic evidence are of little value for diagnosing atypical CAP. Early application of mNGS detection increased the atypical pathogenic identification, improved the prognosis and made up for the deficiency of conventional detection methods.

## AUTHORS' CONTRIBUTIONS

Ning Zhu, Daibing Zhou and Ruyu Yuan conceived and drafted the manuscript as well as collected the data. Yiminniyaze Ruzetuoheti and Jing Li organized and analysed the figures. Xiujuan Zhang performed the literature search. Shengqing Li designed the study and edited and revised the manuscript. All authors read and approved the final version of the manuscript.

## CONFLICT OF INTEREST STATEMENT

The authors declared no potential conflicts of interest concerning this article's research, authorship and publication.

## ETHICS STATEMENT

This study was conducted according to the principles of the Declaration of Helsinki and approved by Huashan Hospital, Fudan University (NO.KY2016‐396). All research data were anonymously analysed.

## PATIENT CONSENT FOR PUBLICATION

Informed consent for data publication was obtained from patients.

## Data Availability

No additional data are available.
